# Synchronous colonic carcinomas presenting as an inguinoscrotal hernial mass: a case report

**DOI:** 10.1186/1752-1947-1-36

**Published:** 2007-06-28

**Authors:** Siao Pei Tan, Siong-Seng Liau, Shayma'u M Habeeb, Dermot O'Riordan

**Affiliations:** 1 Department of General Surgery, West Suffolk Hospital, Hardwick Lane, Bury St Edmunds, IP33 2QZ Suffolk, UK

## Abstract

**Background:**

A carcinoma within a hernia in the groin is uncommon, with an incidence of less than 0.5 percent of all excised sacs. This article describes a case of synchronous colonic carcinomas, one of which presented as an inguinoscrotal mass.

**Case presentation:**

A 69-year old man presented with a large, irreducible left inguinoscrotal hernia and symptoms of obstruction. On examination, there was an 8 cm palpable mass within the hernia sac. CT scan revealed small and proximal large bowel obstruction secondary to a large ingunoscrotal sac and synchronous colonic tumours of the transverse colon and the ascending colon. The former presented as an inguinoscrotal mass. Laparotomy revealed a large tumour mass arising from the transverse colon in the hernia sac. The procedure was followed by an extended right hemicolectomy, during which the second tumour in the ascending colon was also resected.

**Conclusion:**

This case demonstrates a rare but interesting occurrence of primary transverse colon carcinoma presenting in a hernia sac, in conjunction with a synchronous tumour of the ascending colon. Prognosis is comparable to patients with a solitary tumour of similar pathological staging when the resection is curative. The presence of an inguinal hernia itself does not signify an increased risk of colorectal malignancy. However, in the presence of obstruction, incarceration, and weight loss, malignancy should be suspected. Thorough clinical examination, flexible sigmoidoscopy or radiographic evaluation is necessary preoperatively in such patients. Surgical resection, with or without adjuvant oncological treatment, should be performed as soon as possible, using established techniques with modifications according to involvement of local structures.

## Background

Carcinomas in hernias in the groin are divided into saccular, intrasaccular and extrasaccular [1], based on the anatomical relation to the sac. A saccular tumour is when the primary or metastatic disease directly involves the peritoneal sac, (for example a mesothelioma or peritoneal metastases from other organs). Intrasaccular tumour occurs when the incarcerated hernia contains an organ with a primary carcinoma. The commonest of these cases is a sigmoid colon carcinoma presenting in the left inguinal hernia [2]. Hernia contents of urological and gynaecological origin are also possible. We report the first case reported in the literature with one of two synchronous primary tumours presenting within a hernia, and to our best knowledge, the first description of a primary tumour of the transverse colon presenting in an inguinal hernia.

## Case presentation

**Figure 1:**
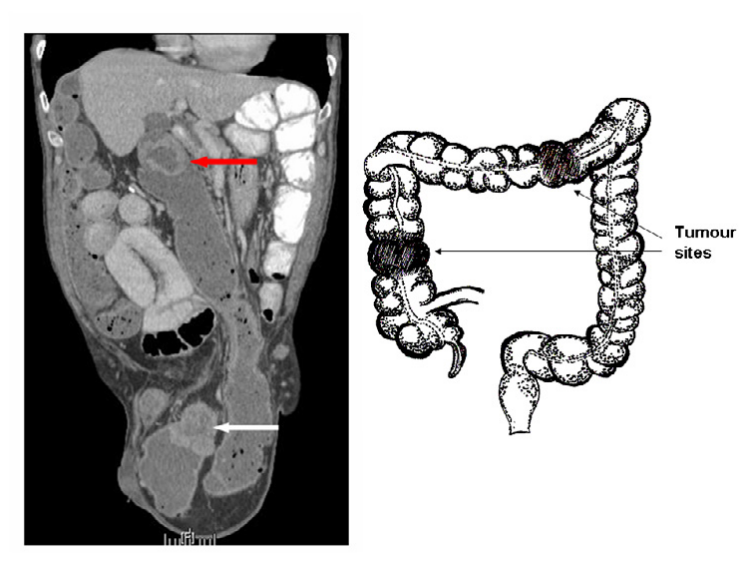
**Imaging of the abdomen (coronal view)**. Left: CT scan showing tumours of the ascending colon, seen at the hepatic flexure (red arrow), and of the transverse colon (white arrow), seen in the left inguinoscrotal hernia sac. Right: Diagrammatic representation of the tumour sites.

**Figure 2:**
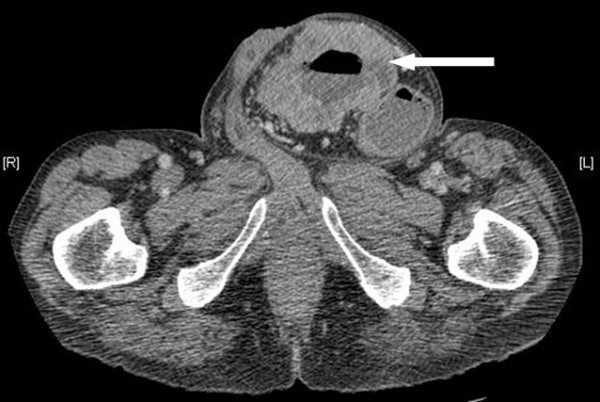
**Imaging of the abdomen (cross sectional)**. CT scan showing tumour of the transverse colon (white arrow) presenting within a left inguinoscrotal hernia.

**Figure 3:**
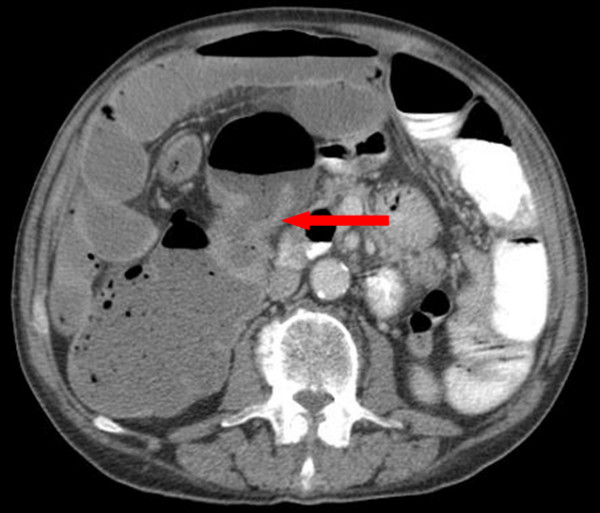
**Imaging of the abdomen (cross sectional)**. CT scan showing tumour of the ascending colon (red arrow).

A 69-year old man with a long-standing irreducible left inguinoscrotal hernia presented with 6–8 weeks history of constant dull ache in the paraumbilical region and associated vomiting. He also reported having loose stools and weight loss of 2 stone over the last 6–8 weeks. On examination, there was a large irreducible left inguinoscrotal hernia with a palpable mass measuring approximately 8 cm within the hernia sac. Plain abdominal film revealed evidence of subacute small bowel obstruction. A subsequent CT revealed small and proximal large bowel obstruction with a large left-sided inguinoscrotal hernia. In addition, there was a loop of transverse colon, with significant circumferential wall thickening, within the hernia sac (See Figure 1 and 2). There was also a second area of circumferential bowel wall thickening with narrowing seen in the region of the hepatic flexure (See Figure 1 and 3). There were no signs of liver metastases or abdominal lymphadenopathy. A full pre-operative metastatic survey and assessment of the entire colon was not performed as he was acutely unwell with bowel obstruction. At laparotomy, it was evident that there were synchronous tumours in the transverse colon close to the splenic flexure and at the ascending colon. The former presented as a mass in an incarcerated left inguinoscrotal hernia. The hernial sac was reduced following release of the external oblique with a groin incision.

## Surgical techniques

Under general and epidural anaesthesia, the patient was placed in the supine position. A midline laparotomy was performed and revealed grossly distended small and large bowel. After an attempt at reducing the incarcerated left inguinoscrotal hernia failed, an incision was made at the left groin to release the external oblique and the hernia was reduced without breach of the hernial sac. The groin incision allowed visualisation of the cord structures and there were no gross signs of tumour invasion locally. A large tumour mass arising from the transverse colon was found in the hernia sac. A further tumour was found at the ascending colon. The procedure was followed by an extended right hemicolectomy with primary ileo-colonic anastomosis. Good margins from both tumours were allowed. A 10-cm section of terminal ileum was excised. Small bowel with interloop adhesion was dissected and freed. The terminal ileum was anastomosed to the descending colon with TLC 75 staples to form a side-to-side functional end-to-end anastomosis.

Histological examination confirmed a moderately differentiated adenocarcinoma (pT3 N0 Mx; Dukes B) of the ascending colon with one focus of extramural vascular invasion. The second tumour was again a moderately differentiated adenocarcinoma of the transverse colon with one focus of extramural vascular invasion (pT3 N0 Mx; Dukes B). All 14 lymph nodes showed no evidence of nodal metastases.

Convalescence was initially complicated by reduced urine output which was managed with fluid balance and use of furosemide. He made a slow but good recovery and was discharged on day 37. The case was discussed in a multidisciplinary meeting. In view of the vascular invasion, a post-discharge oncology outpatient appointment was arranged to discuss the option of adjuvant chemotherapy. He will also be offered left sided colon imaging, either colonoscopy or flexible sigmoidoscopy, to assess the remainder of the colon.

## Discussion

The incidence of synchronous malignancies of the colon and rectum varies from 2 to 11 percent. We need to detect synchronous malignancies, if any, during resection of the index lesions in order to avoid repeated surgery in the future, at which time the tumours are more likely to be of advanced stage and thus bear a less favourable prognosis. We can do this by performing preoperative total colonoscopy, palpating the entire colon intraoperatively, and carefully inspecting the resected segment macroscopically and microscopically after the operation [3].

Most synchronous tumours arise as independent neoplasms. They are generally similar to single lesions in clinical characteristics and pathological findings [3]. However, one study has shown that the male:female ratio was higher and distant metastasis was more frequent in synchronous than in single cases [3]. In a study involving 876 patients where 42 cases (4.8%) were synchronous carcinomas, postoperative survival was significantly shorter in synchronous cases than in single cases on univariate analysis. Nonetheless, in the multivariate proportional hazard model in which pathological stage and curability were included as prognostic co-factors, the difference in postoperative survival between the two groups was insignificant [3]. As such, the prognosis of those with synchronous tumours is similar to those with solitary colon tumours on a stage-for-stage basis when the resections are curative [3] and the highest stage synchronous tumour is considered [4].

There is limited literature on the management of patients with malignancies in hernia sacs and we found no clear evidence on the best approach in treating these patients. The previously reported cases were mainly of sigmoid tumours [5] and to our best knowledge, this is the first reported case of a primary tumour of the transverse colon presenting in an inguinal hernia, in addition to a synchronous tumour at the ascending colon. Intraoperatively, the colonic attachments at the splenic flexure were intact. We speculate that mechanical factors probably played a significant role in the process of herniation of the tumour. It is possible that the tumour served as a point of propulsion and was aided by gravity to herniate at the inguinal region.

Invasion of the contiguous structures within a hernial sac in not unheard of [6]. Lymphatic spread to preaortic nodes via gonadal vessels has been reported, especially when the spermatic cord is involved [6]. This situation warrants a more radical resection and adjuvant oncological treatment. In this present case, through the groin incision, there was no gross evidence to suggest cord involvement. However, in view of the microscopic vascular invasion, the patient was offered a post-discharge oncology outpatient appointment to discuss the option of adjuvant chemotherapy.

The reported increased incidence of colonic malignancies in inguinal hernia patients that exceeds the age-related expected incidence has led some to advocate screening [7]. Gravity [2], raised intra-abdominal pressure secondary to the tumour development, straining on defaecation or partial intestinal obstruction have been said to contribute to the development of hernias. However, studies have found no causative relationship between inguinal hernia and colonic malignancies [8,9]. The current consensus is that patients with inguinal hernias should undergo screening for colon cancer at the same rate as the general population [10]. Nonetheless, a previously reducible hernia with associated symptoms such as obstruction, anaemia, weight loss, or change in bowel habit, should raise a high index of suspicion for colonic malignancies. Investigation such as barium enema, colonoscopy or a CT scan would be appropriate in these situations.

## Conclusion

The present case demonstrates a rare but interesting occurrence of primary transverse colon carcinoma presenting in a hernia sac, in conjunction with a synchronous tumour of the ascending colon. The presence of an inguinal hernia itself does not signify an increased risk of colorectal malignancy. Further, inguinal hernia alone is a relatively rare cause of colonic obstruction. In the present case, the presence of symptoms of obstruction, incarceration, weight loss and a palpable mass within the hernia sac immediately raised the suspicion of malignancy. Thorough clinical examination, endoscopic (e.g colonoscopy) and radiological evaluations (e.g abdominal CT scan) are necessary preoperatively in such patients. Surgical resection, with or without adjuvant oncological treatment, should be performed as soon as possible, using established techniques with modifications according to involvement of local structures. Prognosis is comparable to patients with a solitary tumour of similar pathological staging when the resection is curative.

## Competing interests

The author(s) declare that they have no competing interests.

## Authors' contributions

SPT drafted the article, prepared the illustration and performed the literature search. SSL assisted in performing the surgery, conceived this report, and supervised drafting and revision of the article. SMH helped to acquire the radiological images, prepared the cover letter and performed the literature search. DOR performed the surgery, supervised the drafting and overall structure of the article. All authors have read and approved the final manuscript.
